# Development of right ventricular electromechanical dyssynchrony following surgical repair of tetralogy of Fallot in infants

**DOI:** 10.3389/fped.2024.1443924

**Published:** 2025-01-10

**Authors:** Andrew W. McCrary, Sydney D. Collins, Zebulon Z. Spector, P. Andrea Kropf, Piers C. A. Barker, Joseph Kisslo, Daniel E. Forsha

**Affiliations:** ^1^ Division of Pediatric Cardiology, Department of Pediatrics, Duke University Medical Center, Durham, NC, United States; ^2^ Divison of Cardiology, Department of Medicine, Duke University Medical Center, Durham, NC, United States; ^3^ Division of Pediatric Cardiology, Division of Pediatrics, Children’s Mercy Hospital, University of Missouri-Kansas City, Kansas City, MO, United States

**Keywords:** tetralogy of Fallot, right bundle branch block, electromechanical dyssynchrony, strain echocardiography, dyssynchrony

## Abstract

**Background:**

In adolescents and adults with tetralogy of Fallot (TOF), right ventricle (RV) electromechanical dyssynchrony (EMD) due to right bundle branch block (RBBB) is associated with reduced exercise capacity and RV dysfunction. While the development of RBBB following surgical repair of tetralogy of Fallot (rTOF) is a frequent sequela, it is not known whether EMD is present in every patient immediately following rTOF. The specific timing of the onset of RBBB following rTOF therefore provides an opportunity to assess whether acute RBBB is associated with the simultaneous acquisition of EMD.

**Methods:**

Transthoracic echocardiography with speckle tracking analysis for RV global longitudinal strain (GLS) and 12-lead ECG were performed prospectively on 20 infants following rTOF. Three apical RV views were obtained using analogous imaging planes to the standard LV views to provide a comprehensive evaluation. Regional RV GLS patterns were categorized as synchronous, EMD, or indeterminate. EMD was defined as an early-terminated septal contraction opposed by early stretch and post-systolic peak contraction in the activation delayed RV free wall. An indeterminate pattern was defined as a lack of fully synchronous contraction of all segments but not meeting criteria for EMD. Pre-rTOF echocardiograms and ECGs were analyzed to confirm the presence of synchronous contraction and a normal QRS pattern and duration prior to surgery.

**Results:**

Twenty TOF infants (median age 87 days; 8 days from surgery to post-rTOF evaluation) demonstrated QRSd prolongation following rTOF (pre-rTOF 58 ± 9 ms; post-rTOF 97 ± 14 ms; *p* < 0.001) with new RBBB morphology in all but one patient. All pre-rTOF RV strain patterns were synchronous. Post-rTOF RV strain analysis showed EMD in 25% (5/20) and an indeterminate pattern in 40% (8/20) with the remaining 35% (7/20) maintaining a synchronous pattern, including the patient without RBBB. The EMD group had the lowest RV GLS following repair (*p* = 0.006).

**Discussion:**

Acquisition of acute QRS prolongation in a RBBB pattern is near-universal following rTOF but without matched or identical patterns of dyssynchrony, suggesting that variations in the time from electrical to electromechanical dyssynchrony potentially caused by differences in right bundle branch anatomy and injury may be relevant to electromechanical outcomes.

## Introduction

Progressive right ventricular (RV) failure is a frequent long-term sequela following the complete surgical repair of tetralogy of Fallot (rTOF), contributing to substantial morbidity and mortality as patients move into adolescence and adulthood. The mechanism for this failure was initially presumed to be severe pulmonary valve insufficiency, acquired during the relief of the pulmonary outflow obstruction. The chronic nature of the severe pulmonary insufficiency is then presumed to lead to progressive RV dilation, and eventual RV dysfunction similar to the mechanism of aortic insufficiency leading to left ventricular dysfunction in adults (1). While pulmonary valve replacement to restore pulmonary valve competency in later childhood or early adulthood improves RV dilation, this intervention fails to fully resolve RV dysfunction and decrease mortality (2–4). The persistence of RV dysfunction in the setting of subsequently acquired pulmonary valve competency therefore raises the important question of whether RV dilation is the sole mechanism for dysfunction.

Post-operative right bundle branch block (RBBB) is also a common sequela of rTOF, and its degree of prolongation is independently associated with RV dilation and risk of sudden death (5–7). A growing body of research has noted an association between RV electromechanical dyssynchrony (EMD) in rTOF patients and adverse RV remodeling, worse RV systolic function, decreased exercise capacity, and even atrial arrhythmias independent of the degree of pulmonary valve insufficiency (8–10). The combination of electromechanical dyssynchrony in the setting of coexisting RBBB is present in most adolescent and adult patients after rTOF (9, 11–13). However, it is unclear whether dyssynchrony is present at the onset of development of RBBB in patients with rTOF, or if dyssynchrony develops as its own sequela of chronic RBBB or pulmonary valve insufficiency.

Investigations into left ventricular dyssynchrony using speckle tracking strain analysis in adult patients, specifically identifying and defining classic pattern dyssynchrony (CPD), have proven clinically useful by (1) specifically identifying dyssynchrony due to left bundle branch block; (2) being reproducibly and distinctly different from other uncoordinated strain patterns due to scar, surgical incision, and changes in loading conditions (14); and (3) predicting better response to cardiac resynchronization therapy (CRT) in dyssynchrony-induced heart failure (15, 16). CPD also has been seen in subgroups of patients across the spectrum of congenital and acquired pediatric heart disease (17–20).

Insight into a connection between electrical conduction delay, such as bundle branch block or classic pattern dyssynchrony, and dyssynchrony-induced heart failure has been previously best understood through examining the outcomes of adult patients undergoing transcatheter aortic valve replacement who develop left bundle branch block as an immediate and persistent post-procedural outcome (21). We therefore hypothesize that patients with rTOF will have the similar immediate development of dyssynchrony, but in the *right* ventricle, in association with the acute acquisition of RBBB during the surgical repair.

## Methods

### Study cohort

Pre- and post-operative ECGs and transthoracic echocardiograms with RV strain analysis were compared in 20 subjects from a single center. Inclusion criteria were a diagnosis of TOF with prograde pulmonary blood flow and complete surgical repair in infancy. A complete surgical repair was defined as ventricular septal closure without intentional fenestration and relief of pulmonary outflow obstruction with or without a transannular patch or pulmonary arterioplasty. Patients with TOF-pulmonary atresia and TOF-absent pulmonary valve were excluded, as were TOF patients requiring any preceding palliative procedure or unable to be completely repaired in a single surgery. This study was approved by the Duke Institutional Review Board and informed consent was obtained for all subjects.

### Electrocardiography protocol

12-lead electrocardiograms (ECGs) were performed on all subjects before and after complete repair and reviewed by a pediatric electrophysiologist (ZZS). QRS duration was measured in leads II, V1, and V5, and averaged for the final reported QRS duration. Right bundle branch block was defined as a QRS duration longer than the upper limit of normal for age, with new rightward deviation of the QRS axis, and characteristic QRS morphology in lead V1 (rsR', R, qR, Qr) (22).

### Echocardiography protocol

Transthoracic echocardiograms and RV strain pattern analyses were performed on all 20 subjects before and after complete surgical repair. All echocardiograms were performed free breathing and without inotropic support. All post-surgical imaging and analyses were performed prospectively on a GE Vivid E9 or E95 (GE, Vingmed Ultrasound, Horten, Norway).

**Figure 1:**
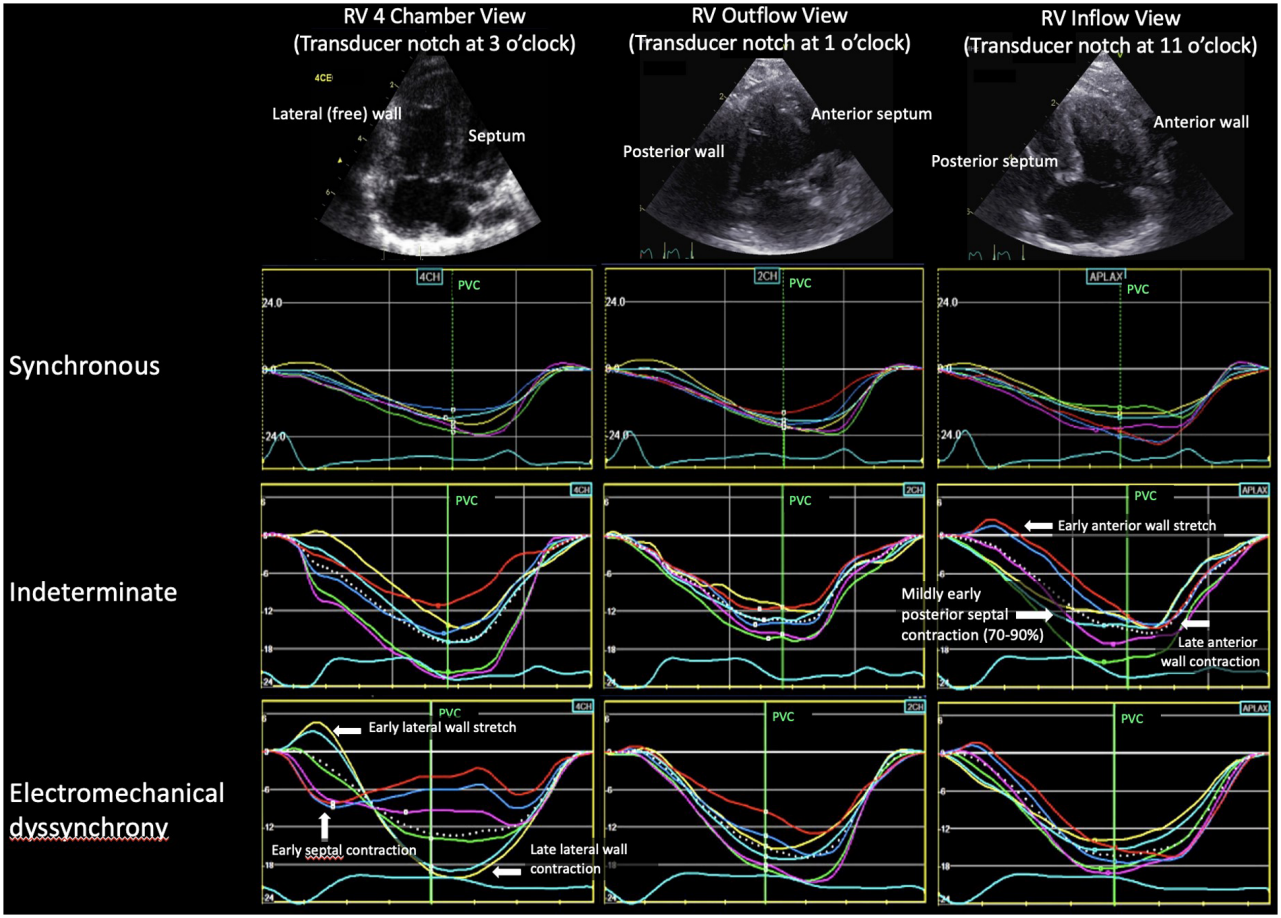
Example of the three strain patterns for each RV view. In the RV 4 chamber and outflow views, the septal strain segments are red, dark blue, purple and the lateral wall strain segments are yellow, light blue, green. Because the notch is at 11 o'clock for the RV inflow view, the colors are reversed. RV, right ventricle; PVC, pulmonary valve closure.

Two-dimensional imaging was optimized for longitudinal 2D speckle tracking strain analysis using a 6 or 12 MHz ultrasound probe from three apical RV views selected to evaluate three different opposing walls for dyssynchrony, analogous to the three standard apical LV views as previously described (23). Examples of these views and the RV walls imaged are shown in Figure 1. These post-operative echocardiograms were compared with pre-operative echocardiograms in all subjects. An identical prospective protocol was used to obtain the pre-operative echocardiograms on subjects enrolled prior to their surgical repair (*n* = 10). For subjects referred from outside institutions and enrolled at the time of surgical repair (*n* = 10), retrospective strain analysis was performed on clinically ordered pre-operative echocardiograms. These retrospective echocardiograms were acquired on a Vivid E9 (GE) or a Phillips IE33 or Epiq imaging system (Phillips Medical Systems, Andover, MA) and had a single apical RV view (RV focused four-chamber view) per the standard clinical protocol. The primary goal of the pre-operative studies was to exclude the presence of any RV electromechanical dyssynchrony prior to surgery.

### 2D RV strain protocol

Strain analysis for all prospective studies was performed off-line in GE EchoPac PC version BT13, while retrospective analysis was performed using vendor neutral software (Tomtec, Image Arena 2D Cardiac Performance Analysis version 1.2, Tomtec Imaging Systems, Unterschleissheim, Germany). The endocardial border was traced in end-systole from the lateral tricuspid valve annulus to the tip of the septal crest (excluding the VSD patch region), and the region of interest was adjusted to exclude the pericardium, papillary muscles, and chordal apparatus. The integrity of speckle-tracking was evaluated by the software and visually confirmed by the reader. In the case of poor tracking, the tracing and shape of the region of interest were adjusted by the reader. Mid-myocardial RV global longitudinal peak strain (RV GLS) was calculated as the average of the GLS from each of the RV views (except for the retrospective cases which were calculated using the four-chamber RV view only). Segments without adequate tracking on repeated attempts were rejected and views with more than two rejected segments were excluded from the analysis.

### Strain pattern analysis

RV longitudinal strain pattern analysis from all studies was analyzed independently by two experienced investigators (DF, JK). For analysis timing, tracings were set to start at the onset of the QRS. In Figure 1, pulmonary valve closure (PVC) relative to the QRS complex was defined on a spectral Doppler tracing of pulmonary outflow. The definitions for strain pattern analysis categories (synchronous, electromechanical dyssynchrony, and indeterminate) are presented in Table 1.

**Table 1:** Definitions of RV strain pattern analysis.

Strain pattern	Definition
Synchronous	• All regional segments contracting together with similar peak strain
Indeterminate pattern^a^	• Early free wall stretch with peak contraction after semilunar valve closure of at least one segment • Mildly early septal peak contraction of at least one segment (70%–100% through systole) that is not early enough to meet criteria for EMD
Electromechanical Dyssynchrony (EMD)^b^	• Early free wall stretch with peak contraction after semilunar valve closure of at least one segment • Significantly early terminated peak contraction of at least one septal segment • The early termination has to be in the first 70% of systole.

^a^ Adapted from Klein et al. Immediate mechanical effects of acute left bundle branch block by speckle tracked strain. *J Electrocardiol*. 2015;48(4):643–651.

^b^ Adapted from Risum et al. Simple regional strain pattern analysis to predict response to cardiac resynchronization therapy: rationale, initial results, and advantages. *Am Heart J*. 2012;163(4):697–704.

### Statistical analysis

Nonprobability consecutive sampling was used for cohort selection. Standard summary statistics were used to compile data including medians with interquartile range or means with standard deviations for continuous variables and percentages for discrete variables. The cohort was summarized by clinical, electrocardiographic, and echocardiographic variables. Observed frequencies and continuous variables were assessed using bivariate testing including *t*-tests and Fisher's exact tests. Comparisons between three groups were analyzed with one-way analysis of variance (ANOVA) test.

## Results

The median age at time of surgery for the 20 TOF subjects was 87 days (IQR 51–150 days). One of two surgeons performed all procedures. All VSDs were repaired via transatrial approach with mostly a bovine pericardial patch (15 of 20; remainder were synthetic polyester fiber patches). Clinical characteristics are summarized in Table 2 with pre- and post-operative strain and ECG measurements summarized in Table 3. Prior to surgery, all subjects had a normal QRS duration, normal RV function and synchronous RV strain patterns. The majority of subjects (15/20) underwent complete TOF surgical repair with a transannular patch; the remainder (5/20) had TOF repair with a valve sparing technique and muscle bundle resection.

**Table 2:** Clinical characteristics.

Clinical Characteristics	All Subjects (20)
Female	9 (45%)
Age at rTOF (days)	87 (51–150)
Weight at rTOF (kg)	5.5 ± 2
Body Surface Area (m^2^)	0.28 ± 0.07
Neonatal rTOF	5 (25%)
Type of rTOF	
• Transannular patch	15 (75%)
• Valve sparing	5 (25%)
Cardiopulmonary bypass time (mins)	158 ± 60
Time from surgery to strain echo (days)	8 (5–13)
Heart rate on post strain echo (BPM)	143 ± 19

*N* (%) or Mean ± SD or Median (IQR).

rTOF, complete repair of tetralogy of Fallot; GLS, global longitudinal peak strain average; SD, standard deviation; IQR, interquartile range; mins, minutes; BPM, beats per minute.

**Table 3:** Pre- and post-operative measures.

Mechanics measurements	Pre-operative	Post-operative	*p* value
Right bundle branch block	0 (0%)	19 (95%)	<0.001
QRS duration (ms)	58 ± 9	97 ± 14	<0.001
RV strain pattern			
• Synchronous • Indeterminate • EMD	20 (100%) 0 (0%) 0 (0%)	7 (35%) 8 (40%) 5 (25%)	
RV GLS (%)^a^	−21.5 ± 3.2	−16.0 ± 3.1	<0.001

^a^ Average for the 10 subjects with pre- and post-operative prospectively protocoled (18 segment GLS) echocardiograms. The 10 subjects who had retrospective pre-operative strain analysis (Tomtec 6-segment) and prospective post-operative strain analysis (EchoPAC, 18-segment) are not included in this GLS comparison due to different strain platforms.

*N* (%) or Mean ± SD or Median (IQR).

EMD, electromechanical dyssynchrony; GLS, global longitudinal peak strain average; IQR, interquartile range; RBBB, right bundle branch block; rTOF, complete repair of tetralogy of Fallot; RV, right ventricle; SD, standard deviation.

Electrocardiograms performed immediately following surgery (postoperative day 0) were abnormal in almost all subjects (19/20, 95%) with a prolongation in QRS duration meeting criteria for RBBB. The final subject met criteria for RBBB with QRS prolongation by 3 months after surgery. Post-operative echocardiograms were performed a median of 8 days (IQR 5–13 days) following surgery. Strain patterns remained synchronous in 7 subjects, newly met criteria for EMD in 5 subjects, or shifted to an indeterminate pattern in 8 subjects as shown in Table 4. The post-rTOF RV GLS was diminished across the entire cohort (RV GLS −16 ± 3.1%) with the most preserved RV GLS present in subjects who maintained synchronous RV mechanics (−17.7 ± 2.9%), followed by the indeterminate group (−16.5 ± 2.4%), and the EMD group (−12.9 ± 2.4%). When comparing the EMD vs. non-EMD groups, EMD was associated with lower RV GLS (*p* = 0.006) while there was no difference in age, weight, surgical repair type, post-rTOF QRS duration, and pre-rTOF RV GLS. No RV views were excluded from analysis due to poor tracking. Both readers independently agreed on the strain pattern in all subjects.

**Table 4:** Characteristics by RV strain pattern.

Clinical Characteristics	RV Strain Pattern			Comparison between 3 groups^a^	Comparison between non-EMD vs. EMD groups^b^
	Synchronous (*n* = 7)	Indeterminate (*n* = 8)	EMD (*n* = 5)	*p* value	*p* value
Female	4 (57%)	3 (38%)	2 (40%)		
Age at rTOF (days)	77 (22–113)	77 (50–113)	157 (135–204)	0.288	0.112
Weight at rTOF (kg)	4.8 ± 1.9	5.5 ± 2.3	6.5 ± 1.5	0.385	0.218
Body Surface Area (m^2^)	0.3 ± 0.07	0.3 ± 0.08	0.3 ± 0.04	0.362	0.196
Neonatal rTOF	3 (43%)	2 (25%)	0		
Type of rTOF					
• Transannular patch	5 (71%)	7 (88%)	3 (60%)		
• Valve sparing with muscle bundle resection	2 (29%)	1 (12%)	2 (40%)	0.558	
Cardiopulmonary bypass time (mins)	166 ± 64	168 ± 64	132 ± 37	0.576	0.288
Surgeon					
• A	3 (43%)	5 (62%)	5 (100%)		
• B	4 (57%)	3 (38%)	0 (0)	0.144	
Time from surgery to echo (days)	8 (7–15)	8 (5–14)	7 (6–12)	0.545	0.476
Heart rate on post-surgery echo (BPM)	147 ± 18	141 ± 19	140 ± 18	0.774	0.755
Post-rTOF RBBB	7 (100%)	7 (88%)	5 (100%)		
Post-rTOF QRS duration (ms)	95 ± 15	95 ± 18	103 ± 4	0.633	0.333
Post-rTOF RV GLS (%)	−17.7 ± 2.9	−16.5 ± 2.4	−12.9 ± 2.4	0.018	0.006

^a^ Comparison by one-way Analysis of Variance for independent samples or Fisher Exact Probability Test.

^b^ Comparison by unpaired *t*-test.

*N* (%) or Mean ± SD or Median (IQR).

RV, right ventricle; EMD, electromechanical dyssynchrony; GLS, global longitudinal strain; IQR, interquartile range; ms, milliseconds; RBBB, right bundle branch block pattern; rTOF, complete repair of Tetralogy of Fallot; SD, standard deviation; mins, minutes; BPM, beats per minute.

Evidence of dyssynchrony (EMD or indeterminate patterns) was most frequently seen in the RV 4-chamber and inflow views (Figure 1). Among the five subjects with EMD, the pattern was seen in both the RV 4-chamber and inflow views in two subjects, only the 4-chamber view in two subjects, and only the inflow view in one subject. Among the eight subjects who developed an indeterminate pattern, dyssynchrony was seen in both the RV 4-chamber and inflow views in six subjects and only in the inflow view in two subjects. Dyssynchrony was not seen in the outflow view.

## Discussion

### Summary

Despite minimal difference in RBBB pattern or QRS duration on surface ECGs, different phenotypes of RV conduction were present immediately following surgical repair of TOF in infants with normal RV synchrony prior to surgery. 25% of subjects met criteria for EMD, with diminished RV function by GLS compared to the rest of the cohort. 40% of subjects demonstrated abnormal RV mechanical activation but failed to meet criteria for EMD. Finally, 35% of subjects maintained synchronous mechanical activation of the right ventricle, though ECG still demonstrated the delayed electrical activation of RBBB. In light of recent literature suggesting that the risk of RV failure in late childhood or early adulthood is more dependent on the presence of RV electromechanical dyssynchrony than pulmonary regurgitation in rTOF, the presence of these distinct patterns, from synchronous to EMD, in subjects immediately following surgical repair provides novel insight into the etiology of this process (8).

### Variations in RV conduction pathways

**Figure 2:**
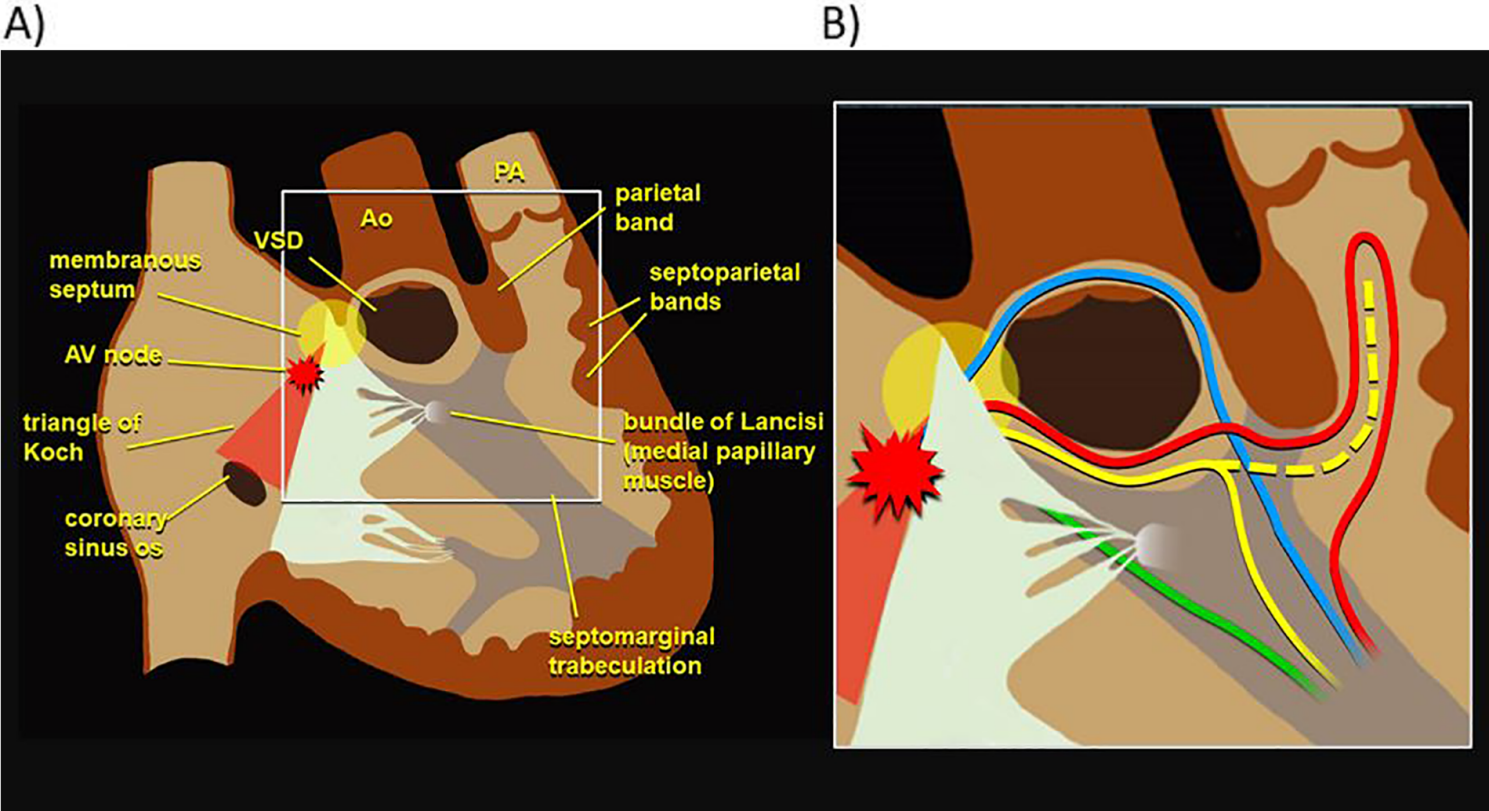
Phenotypic variation in RV electrophysiology. **(A)** Diagram of right ventricular anatomy. **(B)** Various RV conduction phenotypes, with each color depicting a different possible pathway of the right bundle as reported in previous histological analyses of specimens with tetralogy of Fallot (24). RV, right ventricle.

In electromechanical dyssynchrony, an initial electrical activation delay progresses to electromechanical dyssynchrony over time, and the location of right bundle branch injury may affect this progression. Previous histological analyses of the conduction system in specimens with TOF have identified a variety of RV conduction phenotypes, depicted in Figure 2, which vary in their pathways around the ventricular septal defect and through different muscle bundles (24). These variations in conduction patterns as well as in surgical approach for rTOF may result in different levels of injury to the right ventricular conduction system, giving way to different patterns of RV electromechanical activation. Supporting this hypothesis, work from the 1970s and 1980s with epicardial and endocardial mapping of pre- and post-surgical repair of ventricular septal defects (mostly TOF) demonstrated that different sites of electrical block lead to variation in RV activation, despite a similar RBBB pattern on surface ECGs (25, 26).

### Location of right bundle branch injury

Recognizing the variation in right bundle branch patterns as well as surgical techniques may yield insight into the different electromechanical phenotypes seen postoperatively. A more proximal injury to the right bundle, presumably near the site of the ventricular septal defect may produce a delayed but more synchronous mechanical contraction as the ventricle is activated through cell-to-cell activation. In contrast, preservation of the proximal right bundle with injury to a distal ramification of the bundle could lead to differential timing of ventricular activation and thus mechanical dyssynchrony. Previous work has shown that injury in a portion of the terminal ramifications of the right bundle through outflow tract muscle bundle resection and/or ventriculotomy would result in delay in segments of RV free wall with intact and unchanged condition to the apex and septum (27–30). All subjects in this study had either right ventriculotomies or significant muscle bundle resection, but electrical mapping was not performed, so they cannot be differentiated based on our data. However, differences in the site of the bundle injury may explain why some subjects retained a synchronous contraction pattern in the setting of electrical delay while others clearly met criteria for EMD. A future study combining electrical mapping of the right bundle branch with echocardiographic strain analysis of RV mechanics in infants undergoing TOF repair will provide important additional insights into this process.

### Effects of electromechanical dyssynchrony

RV prestretch duration and basal lateral-midseptal delay have been shown to be independently associated with RV dysfunction in rTOF patients, and those same maladaptive contraction patterns, which are also present in patients with Ebstein's anomaly, have correlated with worse RV function and exercise tolerance (9, 31). This suggests that the activation patterns identified in this study may hold predictive value for clinical outcomes in this patient population. Moreover, ventricular-ventricular interactions must be considered when analyzing the effects of RV dyssynchrony, as RV dysfunction leads to LV functional abnormalities, emphasizing the importance of preserving synchronous mechanical contraction even in the setting of global electrical delay in rTOF (10, 32, 33). Although post-operative ECGs showed comparable patterns of RBBB in 95% of subjects in this study, the variable timecourse of progression to electromechanical dyssynchrony is demonstrated with only 25% of subjects meeting criteria for EMD on the post-operative study. In fact, mechanically synchronous contraction patterns were maintained post-operatively in 35% of subjects, despite clear electrical delay on ECG. The remaining 40% of subjects demonstrated altered mechanical contraction patterns on strain analysis after surgical repair that did not fully meet criteria for EMD. These “indeterminate” patterns may represent a transitional stage during which time the RBBB starts to impact RV mechanics, but the septal contraction is not yet completely terminated by the late freewall contraction. Alternatively, this pattern could represent transient RV dysfunction and dispersion related to perioperative factors and requires further study. Duration from RBBB to complete EMD may vary as it does for LBBB in adults with normal anatomy. With this in mind, perhaps the surgical techniques for repairing TOF ought to be reevaluated, since they have remained relatively unchanged for the last fifty years despite TOF persisting as the most common cause of cyanotic heart disease in children. For example, intraoperative electrophysiologic mapping could be used to inform surgical planning when repairing TOF. If interference into the RV conduction system is unavoidable during surgical repair, perhaps transecting the conduction pathway proximally, inducing delayed but near simultaneous mechanical activation, would preserve RV synchronous contraction patterns. However, even with RV synchronous mechanical contraction, electrical delay of the RV with respect to the LV may prove to be consequential. A longitudinal study analyzing biventricular synchrony and function in these patients would be needed to better understand the progressive development of electromechanical dyssynchrony. If avoidance of RBBB is not possible, resynchronization therapy may be a useful intervention for RV electromechanical dyssynchrony, as it has demonstrated consistent improvements in left ventricular ejection fraction and QRS duration in patients with rTOF (34).

### Limitations

In this study, the relatively small sample size did not provide adequate power to detect differences that may have clinical and predictive importance for these infants. Not all pre-operative echocardiograms were performed with a prospective protocol on the GE platform. As the primary goal for the pre-operative echocardiograms was to confirm RV synchrony on the strain pattern analysis prior to surgery, this does not impact the study's aim, and all post-repair studies were prospectively protocoled studies. However, this did limit our ability to compare pre- and post-rTOF GLS; any report of GLS data directly comparing pre- and post-repair values are confined to the 10 subjects enrolled prior to surgical repair who had prospectively protocoled studies at both time points (Table 3). Further, this study relied on pattern analysis from experienced imagers. Time to peak data to assess mechanical dispersion was not captured, but should be considered for future investigations into similar cohorts. Additionally, in the subjects who appeared to maintain synchronous contraction patterns on RV strain analysis, it is possible that there is dyssynchrony present that was simply not well visualized from these views. Lastly, as there was only a single post-operative time point, we were not able to comment on the evolution of the patterns over time or the association of EMDwith progressive RV failure and clinical symptoms seen in older TOF patients.

## Conclusions

Though electrocardiographically similar, various patterns of RV mechanical activation were seen immediately following TOF repair. These activation patterns may reflect right bundle variation as well as surgical technique. Given the known progression to RV dysfunction and failure in TOF patients with electromechanical dyssynchrony, renewed investigation is needed into the preservation of the right bundle or its prescriptive transection.

## Data Availability

The raw data supporting the conclusions of this article will be made available by the authors, without undue reservation.
